# *In vitro* inhibition of porcine cytochrome P450 by 17β -estradiol and 17α-estradiol

**DOI:** 10.2478/v10102-011-0014-x

**Published:** 2011-06

**Authors:** Galia Zamaratskaia, Martin Krøyer Rasmussen, Isabelle Herbin, Bo Ekstrand, Vladimir Zlabek

**Affiliations:** 1 Department of Food Science, BioCenter, Swedish University of Agricultural Sciences, SE-750 07, Uppsala, Sweden; 2 Department of Food Science, Faculty of Agricultural Sciences, Aarhus University, Denmark; 3 South Bohemian Research Center of Aquaculture and Biodiversity of Hydrocenoses, Faculty of Fisheries and Protection of Waters, University of South Bohemia in Ceske Budejovice, 389 25, Vodnany, Czech Republic

**Keywords:** pig, liver, microsomes, cytochrome P450, steroid hormones

## Abstract

Sexually mature pigs are known to possess high concentrations of testicular steroids, which have been shown to change the activities of cytochrome P450 *in vitro*. The aim of the present study was to evaluate the regulation of CYP1A and CYP2E1 activity by the steroids dihydrotestosterone (DHT), 3β-androstenol, 17β-estradiol and 17α-estradiol. Catalytic activities of 7-ethoxyresorufin O-deethylase (EROD) and 7-methoxyresorufin O-demethylase (MROD) were used as markers of CYP1A activities, while p-nitrophenol hydroxylase (PNPH) was used as a marker of CYP2E1 activities. Of the steroids tested, only 17β-estradiol and 17α-estradiol inhibited EROD and MROD activities. This inhibition was observed when a steroid concentration of 100 µM was used, while lower concentrations showed no inhibitory effect. PNPH activities were inhibited only by 100 µM of 17β-estradiol. The significance of these results in vivo is unknown because inhibition was only found when concentrations of estrogens higher than physiological levels were used. Nevertheless, the results provided further evidence on the important role of estrogens in regulation of porcine cytochrome P450 activities.

## Introduction

Cytochrome P450 (CYP450) is a large superfamily of mixed-function oxidases found in all living organisms, from bacteria to mammalian species. Their substrates include a large number of endogenous and exogenous compounds such as fatty acids, steroids, organic solvents and drugs. The interest in porcine CYP450 is increasing because pigs can be used as an animal model in pharmacological and toxicological investigations of new drugs and chemical compounds. It is well known that testicular steroids affect porcine CYP450 activity either directly (Zamaratskaia *et al*., 2007; Rasmussen *et al*., 2010, Rasmussen *et al*., 2011a) or by affecting CYP gene/protein expression (Doran *et al*., 2002; Tambyrajah *et al*., 2004; Gillberg *et al*., 2006; Kojima *et al*., 2010). This in turn can modify the presence of foreign compounds or the magnitude by which they are metabolised.

CYP1A is well characterised in many species due to its important role in detoxification (Gonzalez, 2001). Apart from that, CYP1A is known to be involved in the production of highly reactive and toxic metabolites. Several substrates such as persistent organic pollutants induce CYP1A expression through the activation of the aryl hydrocarbon receptor. In pigs, testicular steroids were shown to regulate CYP1A activities by decreasing mRNA expression (Kojima *et al.* 2008, Kojima *et al.* 2010). A large number of substrates are commonly used to distinguish between the activities of CYP isoforms, however their specificities are often debated. CYP1A1 and CYP1A2 activities were in this study estimated as the rate of 7-ethoxyresorufin O-deethylation and 7-methoxyresorufin O-demethylation, respectively.

CYP2E1 is extensively studied in humans for its involvement in the metabolism of ethanol and other toxicologically important compounds (Gonzalez, 2007; Lu and Cederbaum, 2008). We recently demonstrated that CYP2E1 activity was reduced in the presence of testicular steroids in the liver *in vitro* models (Rasmussen *et al*., 2010, Rasmussen *et al*., 2011a). To study CYP2E1 activities, the rate of p-nitrophenol hydroxylation was used. Apart from the toxicological importance, inhibition of CYP450 activity has implications in meat science because porcine CYP1A and CYP2E1 are enzymes responsible for biotransformation of the boar taint compound skatole (Zamaratskaia and Squires,^2009^).

*In vitro* models of the liver are a commonly used tool to study CYP450 enzymes. Such models, particularly hepatic microsomes, are used in investigations of potential activity of inhibitors of the specific CYP450 isoform. In our previous studies, we investigated effects of 17β-estradiol, estrone, androstenone and 3β-androstenol on porcine EROD, MROD and p-nitrophenol hydroxylase (PNPH) activities using a microsomal preparation (Rasmussen *et al*., 2010). In the present *in vitro* study, we further evaluated the regulation of CYP1A and CYP2E1 activities. The testicular steroids dihydrotestosterone (DHT), 3β-androstenol, 17β-estradiol and 17α-estradiol were evaluated as potential inhibitors of porcine liver CYP activity.

## Materials and methods

### Chemicals

Resorufin, 7-ethoxyresorufin, 7-methoxyresorufin, p-nitrophenol, 4-nitrocatechol, DHT, 17β-estradiol, 17α-estradiol and reduced β-nicotinamide adenine dinucleotide phosphate (NADPH) were obtained from Sigma-Aldrich (Steinheim, Germany). 3β-androstenol was purchased from Steraloids, Inc. (USA). Acetonitrile and methanol of HPLC grade were purchased from Merck (Darmstadt, Germany). A stock solution of the steroids (5 mM) was prepared in methanol and stored at +4°C, until it was used for microsomal incubations.

### Animals and sampling

The microsomes for studying EROD and MROD activity were prepared from liver samples obtained from three entire male pigs of a crossbreed between Yorkshire×Landrace. The pigs were born and raised at the experimental station Funbo-Lövsta at the Swedish University of Agricultural Sciences. The microsomes for PNPH activity were prepared from liver samples obtained from three entire male pigs of a crossbreed between Landrace×Yorkshire and Duroc. The pigs were born and raised at Lundemosegaard, Denmark. Liver samples were taken at slaughter, immediately frozen in liquid nitrogen and stored at –80°C until microsome preparation. The pigs in both groups were mature at slaughter and weighed from 115 to 130 kg.

### Preparation of hepatic microsomes

The microsomal fraction was prepared by the Ca^2+^-aggregation method as described by Rasmussen *et al.* (2011b). Briefly, liver samples were homogenised in 10 mM Tris-HCl, 250 mM sucrose (pH7.4) and centrifuged for 10 minutes at 10.000×g. Afterwards, CaCl_2_ to a final concentration of 8 mM was added to the supernatant, and the samples were centrifuged at 25.000×g for 30 minutes. The protein concentrations of the microsomal fraction (pellet) were determined with a commercially available kit (Bio-Rad laboratories) according to the manufacturer's instructions and bovine serum albumin was used as standard. The prepared microsomes were diluted to a final protein concentration of 4 mg/ml and stored at –80°C before analysis. All procedures were done on ice.

### Enzyme kinetic analysis and inhibition study

Activities of EROD, MROD and PNPH were determined as previously described (Zamaratskaia & Zlabek, 2009; Zamaratskaia *et al*., 2009). Briefly, microsomes were incubated together with the appropriate type of substrate and NADPH at 37°C. The resulting production of resorufin or p-nitrochetecol was measured by HPLC. For studying inhibition, steroids were additionally added to the microsomes. Inhibition studies were performed using a pool of microsomes from three male pigs. The assays were conducted in the presence of the steroids DHT, 17β-estradiol, 17α-estradiol or 3β-androstenol. The concentrations of the steroids were 0.55, 1.0 and 100.0µM (DHT and 3β-androstenol) or 0.02, 1.0 and 100.0µM (17β-estradiol and 17α-estradiol) in a final reaction volume of 500µl for EROD and MROD, and 250µl for PNPH. All steroids were dissolved in methanol. The final concentration of methanol was below 0.1% in the reaction volumes. The same amount of methanol was added to the blank incubations. For the kinetic studies, the activities were determined with substrate concentrations ranging from 0.1 to 4.0µM for EROD and MROD, and from 0.01 to 2mM for PNPH. Kinetic studies were performed under linear conditions.

Michaelis-Menten parameters, maximum reaction velocity (Vmax) and Michaelis constant (Km) were estimated using a nonlinear regression analysis (GraphPad Prism version 4.0 for Windows, GraphPad Software, San Diego, California, USA). Eadie-Hofstee plots were used to classify the enzyme kinetics as mono- or biphasic, *i.e.* whether one or more enzymes were involved in the reaction. When Eadie-Hofstee plots indicated biphasic kinetics, the following equation was applied to estimate kinetic parameters:

V=(Vmax1*S)/(Km1+S)+(V max2*S)/(Km2+S),

where Km1 and Km2 are Michaelis constants for the high- and low-affinity components, respectively, and Vmax1 and Vmax2 are the maximum reaction velocities for the high- and low-affinity components, respectively.

The activities were measured in pooled microsomes, thus taking into consideration between-individual variations in enzyme activities and potential response to the presence of steroids. The activity was regarded as altered if it differed from the control activity by at least 30%.

## Results

### CYP1A

**Figure 1:**
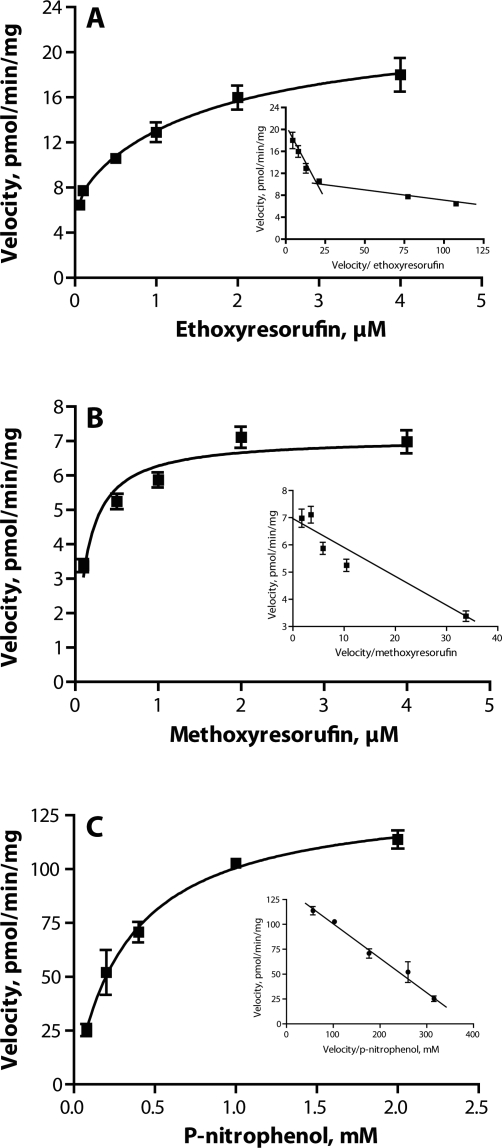
Saturation curve for 7-ethoxyresorufin O-deethylation, EROD (**A**), 7-methoxyresorufin O-demethylation, MROD (**B**) and p-nitrophenol hydroxylation, PNPH (**C**) by porcine hepatic microsomes. The inserts represent the corresponding Eadie-Hofstee plots. Each data point represents the mean of duplicate determinations. Differences between duplicates did not exceed 10% for EROD and MROD, and 15% for PNPH.

**Figure 2:**
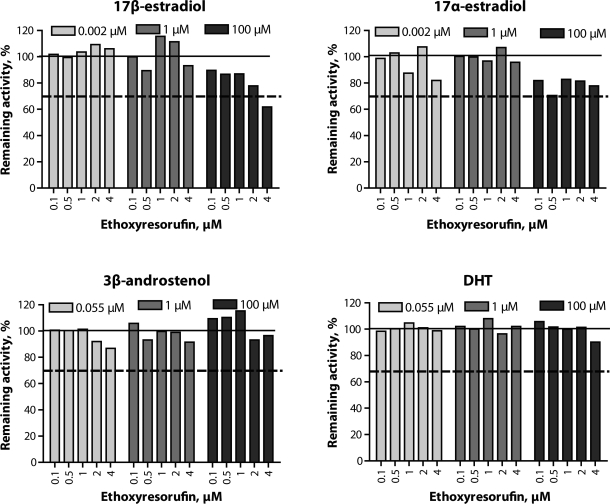
Effect of steroids on 7-ethoxyresorufin O-deethylation in porcine hepatic microsomes. Data are presented as percentage of the enzyme activity in control incubations. Solid line represents activity in control incubations and dashed line represents 70% of the remaining activity. Differences between duplicates did not exceed 10%.

**Figure 3:**
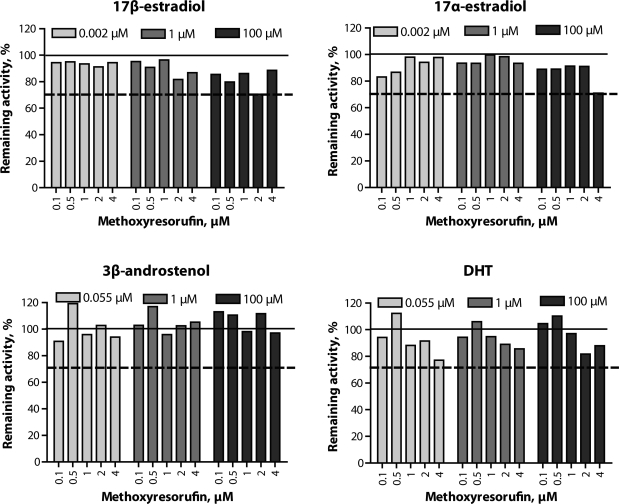
Effect of steroids on 7-methoxyresorufin O-demethylation in porcine hepatic microsomes. Data are presented as percentage of the enzyme activity in control incubations. Solid line represents activity in control incubations and dashed line represents 70% of the remaining activity. Differences between duplicates did not exceed 10%.

Kinetic analysis of the Eadie-Hofstee plots of EROD activities in porcine liver microsomes showed a biphasic response (R^2^=0.884; Figure 1a), whereas MROD activities followed the classical Michaelis-Menten model (R^2^=0.869; Figure 1b). The kinetic parameters for EROD and MROD are shown in Table 1. Of the steroids evaluated in the present study, EROD and MROD activities were inhibited only by estrogens, 17β-estradiol and 17α-estradiol, at the concentration of 100µM (Figures 2 and 3). There were indications of MROD inhibition by DHT (Figure 3), although the magnitude of this inhibition did not exceed 30%.

**Table 1:** Kinetic parameters of resorufin formation from 7-ethoxyresorufin and 7-methoxy-resorufin and of 4-nitrocatechol formation from p-nitrophenol in the microsomes from entire male pigs.

	K_m_, µM (CI)	V_max_, pmol/min/mg (CI)
**EROD, high affinity component**	0.01 (0.001–0.087)	7.5 (0.01–5.07)
**EROD, low affinity component**	1.7 (0.01–5.24)	15.2 (9.64–20.71)
**MROD**	0.13 (0.076–0.185)	7.1 (6.58–7.61)
**PNPH**	0.33 (0.214–0.452)	134.0 (118.3–149.7)

EROD: ethoxyresorufin O-deethylation; MROD: methoxyresorufin O-demethylation; PNPH: p-nitrophenol hydroxylation; CI: 95% confidence interval. The Km and Vmax values and CI were calculated using nonlinear regression analysis with GraphPad Prism program 4.0 kinetic software.

### CYP2E1

**Figure 4:**
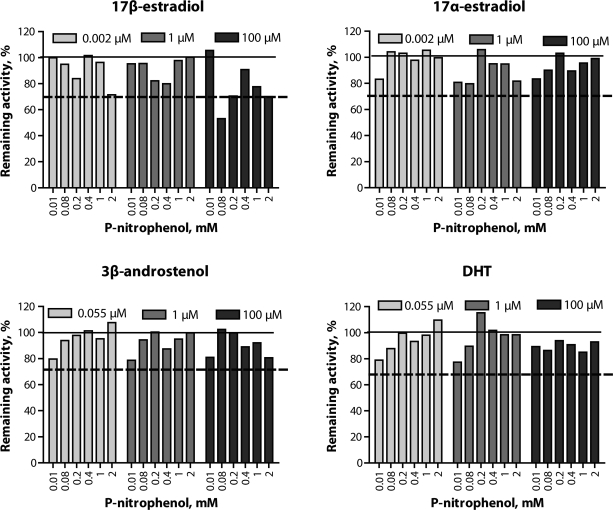
Effect of steroids on p-nitrophenol hydroxylation 7 in porcine hepatic microsomes. Data are presented as percentage of the enzyme activity in control incubations. Solid line represents activity in control incubations and dashed line represents 70% of the remaining activity. Differences between duplicates did not exceed 15%.

The kinetics of PNPH activity in pooled porcine liver microsomes were best described by the Michaelis-Menten model (R^2^=0.968; Table 1). An Eadie-Hofstee plot showed a monophasic response within the substrate concentration range studied (Figure 2C). Inhibition of PNPH activity was observed in the presence of 100µM of 17β-estradiol (Figure 4); apart from that no inhibition occurred.

## Discussion

The knowledge about regulation of CYP450 activity is essential in the investigations of metabolic pathways and pharmacological interactions of xenobiotics. Previously we showed that 15-minute incubation of microsomes with the steroids investigated before adding the substrate (*e.g.* PNPH) increased the inhibitory effect of the steroid, indicating a mechanism-based nature of inhibition (Rasmussen *et al*., 2011a). In the present study, we investigated the kinetic parameters of the CYP's, and consequently the preincubation was omitted from the present inhibition protocol.

Activity of porcine CYP1A and CYP2E1 is regulated by gender (Rasmussen *et al*., 2010), dietary compounds (Rasmussen *et al*., 2011c; Zamaratskaia *et al*., 2005) and the presence of testicular steroids (Rasmussen *et al*., 2010, Rasmussen *et al*., 2011a). In sexually mature pigs, the activity of CYP1A and CYP2E1 has great impact on the accumulation of skatole in adipose tissue. This in turn affects meat quality.

### CYP1A

Numerous studies have used EROD to estimate CYP1A1 activity, while MROD is usually used to estimate CYP1A2 activity (Kojima *et al*., 2008). A study by Zamaratskaia and Zlabek (2009) showed that the two substrates were not entirely specific towards what is usually anticipated.

Plotting the EROD rates found with increasing substrate concentrations in an Eadie-Hofstee plot showed a biphasic course of the curve, indicating that at least two distinct enzymes are responsible for the formation of resorufin from 7-ethoxyresorufin. The resulting curve might, however, also be due to structural changes in enzyme protein molecule. The corresponding Eadie-Hofstee plot of MROD activities showed a monophasic response. This indicates the activity of only one enzyme involved in MROD, or alternatively the involvement of two or more enzymes with similar kinetic parameters. The estimated kinetic parameters obtained for EROD and MROD were similar to those obtained previously using a larger range of substrate concentrations (Zamaratskaia & Zlabek, 2009). The activities of both EROD and MROD were reduced in the presence of estrogens. However, this reduction occurred only when 100µM estrogen were used. This concentration of estrogen is well above what is usually found in pigs (Zamaratskaia *et al*., 2009); thus the significance of those *in vitro* findings for an *in vivo* situation is unknown. The effect of 17β-estradiol on CYP1A activity has previously been investigated in the microsomes from entire and castrated male pigs (Zamaratskaia *et al*., 2009). In that study, the range of the 17β-estradiol tested went from 1.8 to 72pmol/ml, and such concentrations of 17β-estradiol did not alter EROD activity. Correspondingly, the present study showed that low concentrations of 17β-estradiol had no effect on EROD activity.

The intriguing observation in the present study is an involvement of 17α-estradiol in the regulation of EROD and MROD activities. Addition of 100 µM of 17α-estradiol to the incubations reduced the activities of both EROD and MROD. In humans, 17α-estradiol was for a long time considered an inactive isomer of 17β-estradiol. Later, it was shown that 17α-estradiol possessed estrogenic activities, and the number of studies investigating its metabolism and physiological functions keeps increasing (Hobe *et al., * 2002). In pigs, however, the number of studies on this steroid is limited and its physiological concentrations in porcine tissues are unknown. Our study suggests that 17α-estradiol has the potential to inhibit EROD and MROD activities in a way similar to that of 17β-estradiol.

### CYP2E1

An Eadie-Hofstee plot of PNPH activity showed a monophasic response, which indicates that one single enzyme is responsible for this reaction. Skaanild & Friis (2007) suggested that PNPH could be metabolized by both CYP2E1 and CYP2A in pigs. Our present analysis of PNPH rates found with increasing substrate concentrations suggests that only one enzyme is responsible for the metabolism of PNPH, at least at the substrate range used.

Zamaratskaia et al. (2007) reported that 17β-estradiol acted as a mixed inhibitor of PNPH in the microsomes obtained from prepubertal male pigs. In the present study, 17β-estradiol inhibited PNPH activity in the microsomes from mature male pigs although with a lower magnitude of inhibition. The ability of physiological concentrations of 17β-estradiol to inhibit PNPH activity was recently demonstrated by Rasmussen *et al.* (2010a). Generally, microsomes from prepubertal pigs have a greater capacity to metabolize CYP2E1 substrate compared with older pigs (Zamaratskaia *et al*., 2006). Moreover, our recent work has demonstrated that *in vivo* CYP2E1 activity is negatively related to the levels of testicular steroids (Zamaratskaia *et al., * 2008; Zamaratskaia *et al., * 2009). In this study, we used microsomes from mature pigs. The supposedly long exposure to a high level of testicular steroids *in vivo* might lead to changes in enzyme structure. This may explain why the inhibition of PNPH activity by 17β-estradiol was observed only in the presence of high concentrations of the inhibitor.

## Conclusions

The testicular steroids 17β-estradiol and 17α-estradiol inhibited EROD and MROD activities. However, this inhibition was observed when a steroid concentration of 100 µM was used, which is much higher than the physiological levels of estrogens. PNPH activities were inhibited only by 100 µM of 17β-estradiol. The significance of these results in an *in vivo* situation is unknown because steroid concentrations above physiological levels were used and because of the low magnitude of inhibition. However, the results provided further evidence on the important role of estrogens in direct regulation of porcine cytochrome P450.
